# Modified Bekenstein Hawking Entropy of Five-Dimensioned Static Multi-Charge AdS Black Holes in Gauged Supergravity Theory

**DOI:** 10.3390/e28030335

**Published:** 2026-03-17

**Authors:** Cong Wang, Shu-Zheng Yang

**Affiliations:** 1 Shandong Key Laboratory of Space Environment and Exploration Technology, College of Physics and Electronic Engineering, Qilu Normal University, Jinan 250300, China; 2 College of Physics and Astronomy, China West Normal University, Nanchong 637002, China; szyangcwnu@126.com

**Keywords:** Lorentz breaking, quantum perturbation theory, higher-dimensional black hole spacetime, modified Bekenstein-Hawking entropy

## Abstract

Considering the dynamics of spin-1/2 fermion in higher-dimensional static multi-charge black holes in gauged supergravity theory, taking into account Lorentz breaking and quantum perturbation theory, this study investigates new expressions for the Hawking temperature and Bekenstein-Hawking entropy of such black holes based on WKB theory and quantum tunneling radiation theory, as well as the laws of black hole thermodynamics. The physical significance of the research methods used in this paper and the related results obtained are analyzed. Furthermore, an in-depth discussion is provided regarding the implications of the research content for addressing relevant issues in high-dimensional curved spacetime.

## 1. Introduction

Lorentz dispersion relations are considered a fundamental relationship in modern physics. Both general relativity and quantum field theory are built upon this relationship. However, research into quantum gravity theories suggests the possibility of Lorentz invariance breaking. In flat spacetime, Lorentz symmetry is a global symmetry. In curved spacetime, Lorentz symmetry becomes an approximate symmetry which emerges at low energies and is violated at higher energies. This means that situations arise where Lorentz symmetry is broken. Although a successful theory for an optimal Lorentz dispersion relation in high-energy domains has yet to be established, it is widely believed that the magnitude of this correction term should be on the order of the Planck scale [1,2,3,4,5]. Studying the effects of Lorentz breaking will open a window for us to explore new physical knowledge. Since Lorentz invariance is the foundation of the microscopic physical standard model and general relativity, experimental research on Lorentz symmetry breaking is correspondingly divided into two categories: the particle physics standard model in flat spacetime and general relativity in curved spacetime [6,7,8,9,10,11,12]. In terms of theoretical research, since Einstein’s general relativity is a renormalization gravitational theory, a series of gravitational theories have been proposed in an attempt to seek a pathway to quantum gravity theory. One approach to constructing gravitational theories posits that Lorentz symmetry may break down at high energies, while restoring at low energies to align with current scientific experimental results. For example, The Einstein-aether is a modification of Einstein’s general relativity. This theory introduces kinematic quantities of a unitary time-like vector field, $u^{\mu }$, called the aether, and incorporates it into the gravitational action. The presence of the aether field $u^{\mu }$ defines a preferred time-like direction that violates Lorentz symmetry [13,14,15,16,17,18,19,20]. A series of meaningful studies have been conducted on Einstein’s gravitational theory and modified gravitational theories. Nevertheless, the issue of quantizing gravity remains a topic that requires further in-depth research. Theoretically, there are two aspects of the impact of Lorentz breaking in curved spacetime that deserve attention. The first is to revise the Lagrangian of the full Einstein-aether theory to form the most general diffeomorphism-invariant action for the curved spacetime metric $g_{\mu \nu }$ and the aether field vector $u^{\mu }$ and correct the curved spacetime background due to Lorentz breaking [21,22]. The second aspect is the reasonable modification of the Lagranging of the full Einstein-Aether theory and the action for fermions or bosons in curved spacetimes by introducing targeted Lorentz breaking correction terms [23,24,25,26,27,28,29]. Based on this, variational principles are applied to study the modified forms of the dynamical equations for fermions or bosons, thereby investigating the effects of Lorentz breaking in different curved spacetimes [30,31,32]. The following research content is related to the methods of the second aspect of this study. There have been some reports on the impact of Lorentz breaking on the Hawking temperature and Bekenstein-Hawking entropy of black holes in four-dimensional curved spacetime, as well as *ℏ* perturbation theory [31,32,33]. However, there has yet to be in-depth reporting on the modifications to the dynamical equations for fermions or bosons due to Lorentz breaking in higher-dimensional curved spacetimes, along with corresponding studies on the quantum tunneling radiation characteristics of black holes. Therefore, the main objective of this paper is to present a treatment scheme for Lorentz breaking in the context of quantum tunneling radiation from higher-dimensional black holes and discuss the scientific significance of the conclusions drawn. Regarding high-dimensional spacetimes, since Kaluza and Klein proposed the addition of a compact extra spatial dimension on the basis of four-dimensional spacetime, research has been conducted on related topics such as 11-dimensional supergravity theory (M-theory) and the five types of superstring theories in 10 dimensions. There have been relevant reports on charged black hole solutions, black hole solutions with both electric and magnetic charges, and rotating charged dual-charge black hole solutions within Kaluza-Klein (K-K) theory [34,35,36,37,38,39,40,41]. The spacetime metric of higher-dimensional black holes can take various forms. In order to illustrate the effects of Lorentz breaking on the Hawking temperature and Bekenstein-Hawking entropy of higher-dimensional black holes, as well as demonstrate the research methods and physical significance of the results obtained from the study of quantum tunneling radiation corrections for higher-dimensional black holes, we investigate the thermodynamic characteristics of the 5-dimensional static multi-charge Ads black hole in gauged supergravity theory. This black hole possesses three independent electric charge parameters. By studying the modifications to the Hawking temperature of this black hole due to Lorentz breaking, we delve into the corrected results for the Bekenstein-Hawking entropy and their significance.

The structure of this paper is divided into four sections. In Section 2, we introduce the modified form of the dynamical equation for spin-1/2 fermion. Section 3 discus ses the thermodynamic evolution characteristics of 5-dimensional static multi-charge black holes in supergravity theory and presents the quantum correction results for their Bekenstein-Hawking entropy. The final section provides an in-depth discussion of the research methods and the results obtained.

## 2. Modifications to the Dynamical Equation of Spin-1/2 Fermion in Higher-Dimensional Static Spacetime Due to Lorentz-Breaking

The first known Lorentz breaking term is the Carroll-Field-Jackiw (CFJ) term, which was proposed and explicitly calculated in relevant papers [25]. The modification of the action for spinor fields due to Lorentz breaking should include the CFJ term, the Einstein-aether term, and the chiral term. In four-dimensional flat spacetime, there is a higher-derivative extension of spinor quantum electrodynamics (QED) in 4D, with the action as follows [23,24]:

(1) $$\begin{array}{c} \bar{S}=\int d^{4}x\bar{\psi }\left\{i\gamma^{m}D_{m}\left[1-\alpha \frac{{\left(\gamma^{m}D_{m}\right)}^{2}}{{\bar{m}}^{2}}\right]+\bar{b}\gamma_{5}+\xi {\left(d\cdot D\right)}^{2}-\bar{m}\right\}\psi \end{array}$$

where $D_{m}=\partial_{m}-ieA_{m}.$$\bar{\psi }$ is the complex conjugate of ψ. The coefficient of the CFJ correction term is α, the coefficient of the Einstein-aether correction term is ξ, the coefficient of the chiral correction term is $\bar{b}$, and $\bar{m}$ is the mass of a particle. In higher-dimensional curved spacetime, for example, in *N*-dimensional (N>4) spacetime, one can consider N=n+2 dimensions (n>2). When modifying the action of spin-1/2 fermion, it is necessary to take into account the operational rules of Riemannian geometry as well as the rules for free indices and index contraction in general relativity. The Einstein-aether theory is general relativity coupled with a unit time-like vector field $u^{\mu }$. This theory can be regarded as an effective description of Lorentz breaking in the gravity sector. When considering the unit and timelike vector $u^{\mu }$, the corresponding gravitational action in Einstein-aether theory includes a term related to the Lagrangian of the aether field. The Lagrangian is associated with $g_{\mu \nu }u^{\mu }u^{\nu }$. According to studies on Einstein-aether black holes [24,42,43], these investigations reflect the corrections due to Lorentz breaking in a curved spacetime background. Moreover, taking Lorentz breaking into account, we can add a Lagrangian modification term of the aether field to the action of spin-1/2 fermions in a given curved spacetime background. At the same time, since the spinor field exhibits chirality, the action of spin-1/2 fermions in the given curved spacetime should include a chiral modification term. The representation of the spin-1/2 fermion wave function is related to the matrices and space-time metric $g^{\mu \nu }$ is related to gamma matrices; therefore, the Lagrangian modification term in the particle action also contains the CFJ term. In a given higher-dimensional curved spacetime, we must appropriately modify the action of the spinor field according to Lorentz breaking. This modification should include CFJ correction terms, Einstein-aether correction terms, and chiral correction terms. We propose that the expression for the action of spin-1/2 fermion in higher-dimensional spacetime, after Lorentz breaking corrections, is given by [23,24,31,32,33]:

(2) $$\begin{array}{cc} S & =\int d^{n+2}x\sqrt{-g_{\left(n+2\right)}}\tilde{\psi }\left[i\gamma^{\mu }D_{\mu }\left(1-\frac{a_{0}}{{\tilde{m}}^{2}}\hbar^{2}\gamma^{\mu }\gamma^{\nu }D_{\mu }D_{\nu }\right)+\frac{b_{0}}{\hbar }\gamma^{5}+\frac{c_{0}}{\tilde{m}}\hbar^{2}u^{\mu }u^{\nu }D_{\mu }D_{\nu }-\frac{\tilde{m}}{\hbar }\right]\psi \\ \; & =\int d^{n+2}x\sqrt{-g_{\left(n+2\right)}}L_{f} \end{array}$$

where both $\gamma^{\mu }$ (or $\gamma^{\nu }$) and $\gamma^{5}$ are (n+2)-dimensional Hermitian matrices. The wave function ψ can represent any spin fermions. For clarity, we can consider ψ as the wave function of a spin-1/2 fermion and $\tilde{\psi }$ as the complex conjugate of ψ. $D_{\mu }=\partial_{\mu }+\frac{ieA_{\mu }}{\hbar }+i\Omega_{\mu }$ represents the covariant derivative in curved spacetime, where $\Omega_{\mu }$ is the spin connection and *e* is the charge of the spin-1/2 fermion. The Einstein-aether gravity theory, resulting from local Lorentz breaking, is a general relativity coupled to a dynamical time-like vector field $u^{\mu }$, referred to as “aether.” In this gravitational theory, Lorentz symmetry is broken down only to a rotation subgroup due to the existence of a preferred time direction at every point in spacetime. In Equation (2), the expression $u^{\mu }u^{\nu }$ only yields a physically meaningful calculation when it is combined with the specific spacetime metric and the contraction of free indices is performed using the rules of Riemannian geometry. $u^{\mu }u^{\nu }$ can be expressed using the metric tensor’s raising (lowering) index operations and the contraction (inner product) rules for vectors as $u^{\mu }u^{\nu }=u^{\mu }u_{\nu }g^{\nu \nu }$. For a higher-dimensional spherically symmetric spacetime metric, we have $u^{\mu }u^{\nu }=u^{\mu }u_{\nu }g^{\nu \nu }=u^{t}u_{t}g^{tt}+u^{r}u_{r}g^{rr}+u^{n}u_{n}g^{nn}$. Depending on the characteristics of the spacetime metric, we can appropriately choose $u^{t}$ (or $u_{t}$), $u^{r}$ (or $u_{r}$), and $u^{n}$ (or $u_{n}$) such that the inner product $u^{\mu }u_{\mu }$ is scalar, and we can even make $u^{\mu }u_{\mu }=C$ (constant). $a_{0},b_{0}$, and $c_{0}$ correspond to the coupling coefficients of the CFJ term, chiral term, and Einstein-aether correction term related to Lorentz breaking. All three correction term coefficients are small quantities. $\tilde{m}$ is the mass of the spin-1/2 fermion, and it holds that $\frac{a_{0}}{\tilde{m}}\ll 1,\frac{b_{0}}{\tilde{m}}\ll 1,\frac{c_{0}}{\tilde{m}}\ll 1$. $L_{f}$ is the Lagrangian function. Based on Equation (2) and the variational principle, we can obtain

(3) $$\begin{array}{c} \delta S=\int d^{n+2}x\sqrt{-g_{\left(n+2\right)}}\delta L_{f}=0 \end{array}$$

Therefore, from Equation (2) and Equation (3), we can obtain the following matrix equation [32,33]:

(4) $$\begin{array}{c} \left[i\gamma^{\mu }D_{\mu }\left(1-\frac{a_{0}}{{\tilde{m}}^{2}}\hbar^{2}\gamma^{\mu }\gamma^{\nu }D_{\mu }D_{\nu }\right)+\frac{c_{0}}{\tilde{m}}\hbar u^{\mu }u^{\nu }D_{\mu }D_{\nu }+\frac{b_{0}}{\hbar }\gamma^{5}-\frac{\tilde{m}}{\hbar }\right]\psi =0 \end{array}$$

This is a matrix equation. Its complexity mainly lies in the correct choice of the gamma matrices in (n+2)-dimensional curved spacetime and the accurate representation of the wave function ψ. The WKB theory proposed by Wenzel, Kramers, and Brillouin is a semiclassical approximation theory in which the particle wave function ψ can be represented in terms of the particle action *S*. Here, we utilize WKB theory to express ψ and differentiate the two non-zero components of the contravariant metric tensor $g^{tt}$, $g^{rr}$ and $g^{nn}\left(n>2\right)$ within the high-dimensional static spacetime metric. Therefore, in (n+2)-dimensional spacetime, the wave function of the spinor field ψ can be represented as

(5) $$\begin{array}{c} \psi =\left(\begin{array}{c} A_{\frac{m}{2}\times 1}\left(t,r,\ldots x^{n},\ldots \right)\\ B_{\frac{m}{2}\times 1}\left(t,r,\ldots x^{n},\ldots \right) \end{array}\right)e^{\frac{i}{\hbar }S\left(t,r,\ldots x^{n},\ldots \right)}=\left(\begin{array}{c} A_{\frac{m}{2}\times 1}\\ B_{\frac{m}{2}\times 1} \end{array}\right)e^{\frac{i}{\hbar }S} \end{array}$$

where *S* is the particle action, $A_{\frac{m}{2}\times 1}$ and $B_{\frac{m}{2}\times 1}$ are matrix elements. In (n+2)-dimensional spacetime, the correct choice of gamma matrices is as follows [44]:

(6) $$\begin{array}{cc} \gamma_{m\times m}^{t} & =\sqrt{g^{tt}}\left(\begin{array}{cc} I_{\frac{m}{2}\times \frac{m}{2}} & 0\\ 0 & -I_{\frac{m}{2}\times \frac{m}{2}} \end{array}\right)\\ \gamma_{m\times m}^{r} & =\sqrt{\frac{1}{g_{rr}}}\left(\begin{array}{cc} 0 & I_{\frac{m}{2}\times \frac{m}{2}}\\ I_{\frac{m}{2}\times \frac{m}{2}} & 0 \end{array}\right)=\sqrt{g_{rr}}\left(\begin{array}{cc} 0 & I_{\frac{m}{2}\times \frac{m}{2}}\\ I_{\frac{m}{2}\times \frac{m}{2}} & 0 \end{array}\right)\\ \gamma_{m\times m}^{n} & =\sqrt{g^{nn}}\left(\begin{array}{cc} 0 & i\gamma_{\frac{m}{2}\times \frac{m}{2}}^{j-2}\\ -i\gamma_{\frac{m}{2}\times \frac{m}{2}}^{j-2} & 0 \end{array}\right),3\leq j\leq n+2 \end{array}$$

where $\gamma_{m\times m}^{t}$, $\gamma_{m\times m}^{r}$, and $\gamma_{m\times m}^{n}$ are all Hermitian matrices. For the case of higher-dimensional static spacetimes, the choice in Equation (6) is correct. $I_{\frac{m}{2}\times \frac{m}{2}}$ is the identity matrix of $\frac{m}{2}\times \frac{m}{2}$ and 0 is the zero matrix of $\frac{m}{2}\times \frac{m}{2}$. $\gamma_{\frac{m}{2}\times \frac{m}{2}}^{j-2}$ represents the (j−2)-th gamma matrix of matrix $\frac{m}{2}\times \frac{m}{2}$. Here, $m=2^{(n+2)/2}$ (or $m=2^{(n+1)/2}$) denotes the order of the gamma matrices in even-dimensional (or odd-dimensional) spacetime. In Equation (5), $A_{\frac{m}{2}\times 1}\left(t,r,\ldots ,x^{n},\ldots \right)$ and $B_{\frac{m}{2}\times 1}\left(t,r,\ldots ,x^{n},\ldots \right)$ are column matrices of $\frac{m}{2}\times 1$. Substituting Equation (5) into Equation (4), we obtain

(7) $$\begin{array}{cc} \; & \left\{-\gamma^{\mu }\left(\partial_{\mu }S+eA_{\mu }\right)\left[1+\frac{a_{0}}{{\tilde{m}}^{2}}\gamma^{\mu }\gamma^{\nu }\left(\partial_{\mu }S+eA_{\mu }\right)\left(\partial_{\nu }S+eA_{\nu }\right)\right]-\frac{c_{0}}{\tilde{m}}u^{\mu }u^{\nu }\left(\partial_{\mu }S+eA_{\mu }\right)\left(\partial_{\nu }S+\right.\right.\\ \; & \left.\left.eA_{\nu }\right)+b_{0}\gamma^{5}-\tilde{m}\right\}\left(\begin{array}{c} A_{\frac{m}{2}\times 1}\\ B_{\frac{m}{2}\times 1} \end{array}\right)=0 \end{array}$$

In this equation, the terms containing *ℏ* are neglected. In the next section, we will consider the perturbation of *ℏ*. Therefore, this is a semiclassical matrix equation. Clearly, the matrix Equation (7) is an eigenvalue equation, which can be rewritten as

(8) $$\begin{array}{c} G\left(\begin{array}{cc} I_{\frac{m}{2}\times \frac{m}{2}} & 0\\ 0 & I_{\frac{m}{2}\times \frac{m}{2}} \end{array}\right)\left(\begin{array}{c} A_{\frac{m}{2}}\times 1\\ B_{\frac{m}{2}\times 1} \end{array}\right)=0 \end{array}$$

where $G=-\gamma^{\mu }\left(\partial_{\mu }S+eA_{\mu }\right)\left[1+\frac{a_{0}}{{\tilde{m}}^{2}}\gamma^{\mu }\gamma^{\nu }\left(\partial_{\mu }S+eA_{\mu }\right)\left(\partial_{\nu }S+eA_{\nu }\right)\right]-\frac{c_{0}}{\tilde{m}}u^{\mu }u^{\nu }\left(\partial_{\mu }S+eA_{\mu }\right)$$\left(\partial_{\nu }S+eA_{\nu }\right)+b_{0}\gamma^{5}-\tilde{m}$. Here, each term has no free indices, so *G* is scalar. The condition for the matrix Equation (7) or Equation (8) to have nontrivial solutions is

(9) $$\begin{array}{c} \det\left|\begin{array}{cc} GI_{\frac{m}{2}\times \frac{m}{2}} & 0\\ 0 & GI_{\frac{m}{2}\times \frac{m}{2}} \end{array}\right|=0 \end{array}$$

From this, it follows that we must require G=0; thus, we obtain the equation satisfied by the action *S* as follows:

(10) $$\begin{array}{cc} \; & -\gamma^{\mu }\left(\partial_{\mu }S+eA_{\mu }\right)\left[1+\frac{a_{0}}{{\tilde{m}}^{2}}\gamma^{\mu }\gamma^{\nu }\left(\partial_{\mu }S+eA_{\mu }\right)\left(\partial_{\nu }S+eA_{\nu }\right)\right]-\frac{c_{0}}{\tilde{m}}u^{\mu }u^{\nu }\left(\partial_{\mu }S+eA_{\mu }\right)\left(\partial_{\nu }S+eA_{\nu }\right)+\\ \; & b_{0}\gamma^{5}-\tilde{m}=0 \end{array}$$

We multiply both sides of Equation (10) by $\gamma^{\nu }\left(\partial_{\nu }S+eA_{\nu }\right)$ and neglect the higher-order small quantities $a_{0}^{2},b_{0}^{2},c_{0}^{2},c_{0}b_{0}$. Then, using

(11) $$\begin{array}{cc} \; & \gamma^{\mu }\gamma^{\nu }+\gamma^{\nu }\gamma^{\mu }=2g^{\mu \nu }I\\ \; & \gamma^{\mu }\gamma^{5}+\gamma^{5}\gamma^{\mu }=0 \end{array}$$

we obtain the following equation:

(12) $$\begin{array}{c} \left[g^{\mu \nu }\left(1+2a_{0}\right)+2c_{0}u^{\mu }u^{\nu }\right]\left(\partial_{\mu }S+eA_{\mu }\right)\left(\partial_{\nu }S+eA_{\nu }\right)-b\gamma^{5}-{\tilde{m}}^{2}=0 \end{array}$$

where $b=2\tilde{m}b_{0}.\;$$\gamma^{5}$ is determined by the specific characteristics of the curved spacetime. Equation (12) is a semiclassical equation. It represents the dynamical equation for a spin-$\frac{1}{2}$ fermion with Lorentz breaking corrections in high-dimensional static spacetime. The $u^{\mu }$ or $u^{\nu }$ must be correctly chosen based on the specific features of the spacetime and should satisfy Equation (11). Similarly, $\gamma^{\mu }$ or $\gamma^{\nu }$ and $\gamma^{5}$ must also be correctly selected according to the specific characteristics of the spacetime. The following analysis will focus on the correction effects of Lorentz breaking on the temperature and entropy of such black holes in the context of a specific static multi-charge AdS black hole.

## 3. Modified Entropy of the Five-Dimensional Static Multi-Charge AdS Black Holes in Gauged Supergravity

In this section, we study the general static three-charge Anti-de Sitter (AdS) black hole in five-dimensional STU gauged supergravity theory and its related topics. The spacetime metric of this black hole, Abelian gauge potentials, and scalar fields are [45]

(13) $$\begin{array}{c} ds_{5}^{2}=-\prod_{i=1}^{3}H_{i}^{-\frac{2}{3}}f\;dt^{2}+\prod_{i=1}^{3}H_{i}^{\frac{1}{3}}\left(f^{-1}\;dr^{2}+r^{2}\;d\Omega_{3}^{2}\right) \end{array}$$

where

(14) $$\begin{array}{cc} A^{i} & =\frac{\sqrt{q_{i}\left(q_{i}+2M\right)}}{r^{2}+q_{i}}dt,X_{i}=H_{i}^{-1}\prod_{j}^{3}H_{j}^{\frac{1}{3}}\\ f & =1-\frac{2M}{R}+\frac{r^{2}}{l^{2}}\prod_{i=1}^{3}H_{i},H_{i}=1+\frac{q_{i}}{r^{2}} \end{array}$$

where *M* and $q_{i}$ are the mass and three independent electric charge parameters, respectively, and *l* is the AdS radius. When $q_{i}\neq 0$ and $q_{2}=q_{3}=0$, we have the five-dimensional static charged AdS black hole solution in K-K gauged supergravity. When $q_{1}=q_{2}\neq 0$ and $q_{3}=0$, we obtain the five-dimensional static charged AdS black hole solution in EMDA (Einstein-Maxwell-dilaton-axion) gauged supergravity theory. When $q_{1}\neq q_{2}\neq 0$ and $q_{3}=0$, this corresponds to the five-dimensional static charged AdS Horowitz-Sen black hole solution [46]. When $q_{1}=q_{2}=q_{3}\neq 0$, this is the famous five-dimensional RN-AdS black hole case after the coordinate transformation $\rho^{2}=r^{2}+q_{i}$, etc. Based on Equations (13) and (14), we can choose $u^{t}$ and $u^{r}$ as follows:

(15) $$\begin{array}{cc} u^{t} & =\frac{c_{t}}{\sqrt{g_{tt}}},u^{t}u_{t}=u^{t}u^{t}g_{tt}=c_{t}^{2}\\ u^{r} & =\frac{c_{r}}{\sqrt{g_{rr}}},u^{r}u_{r}=u^{r}u^{r}g_{rr}=c_{r}^{2} \end{array}$$

According to Equation (13), we have $g_{tt}=-\prod_{i=1}^{3}H_{i}^{-\frac{2}{3}}f$, $g^{tt}=\frac{1}{g_{tt}}=-\frac{1}{f}\prod_{i=1}^{3}H_{i}^{\frac{2}{3}}$, $g_{rr}=-\prod_{i=1}^{3}H_{i}^{\frac{1}{3}}f^{-1}$, $g^{rr}=\frac{1}{g_{rr}}=\prod_{i=1}^{3}H_{i}^{-\frac{1}{3}}f$, $g_{nn}=\prod_{i=1}^{3}H_{i}^{\frac{1}{3}}r^{2}$, and $g^{nn}=\frac{1}{r^{2}}\prod_{i=1}^{3}H_{i}^{-\frac{1}{3}}$. The gamma matrices constructed from the equations satisfy Equation (11), which is evident, since we have $\gamma_{m\times m}^{t}\gamma_{m\times m}^{t}+\gamma_{m\times m}^{t}\gamma_{m\times m}^{t}=2g^{tt}I_{\frac{m}{2}\times \frac{m}{2}}$ and $2\gamma_{m\times m}^{r}\gamma_{m\times m}^{r}=2g^{rr}I_{\frac{m}{2}\times \frac{m}{2}}$. To ensure that the chosen $\gamma_{m\times m}^{t}$ and $\gamma_{m\times m}^{r}$ satisfy Equation (11), it is necessary to correctly select $\gamma^{5}$ such that $\gamma^{5}\gamma^{\mu }+\gamma^{\mu }\gamma^{5}=0$. Therefore, based on Equations (13) and (14), we choose $\gamma^{5}$ as follows:

(16) $$\begin{array}{c} \gamma_{m\times m}^{5}=\prod_{i=1}^{3}H_{i}^{-\frac{1}{3}}f\left(\begin{array}{cc} 0 & I_{\frac{m}{2}\times \frac{m}{2}}\\ -I_{\frac{m}{2}\times \frac{m}{2}} & 0 \end{array}\right) \end{array}$$

From Equation (11) and Equation (16), we can derive

(17) $$\begin{array}{c} \gamma^{5}\gamma^{t}+\gamma^{t}\gamma^{5}=0\\ \gamma^{5}\gamma^{r}+\gamma^{r}\gamma^{5}=0 \end{array}$$

It is important to note that the selected $\gamma_{m\times m}^{t},\gamma_{m\times m}^{r},\gamma_{m\times m}^{n},$ and $\gamma_{m\times m}^{5}$ are all Hermitian matrices. Only such a choice can ensure that the physical quantities derived from the study have definite physical significance. In the context of this five-dimensional gauged supergravity static three-charge AdS black hole spacetime, the modified dynamical equation for the spin-$\frac{1}{2}$ fermion is expressed as follows:

(18) $$\begin{array}{cc} \; & -\left[\left(1+2a_{0}+2c_{0}c_{t}^{2}\right)\right]\frac{1}{f}\prod_{i=1}^{3}H_{i}^{\frac{2}{3}}{\left(\frac{\partial S}{\partial t}-eA_{t}\right)}^{2}+\left(1+2a_{0}+2c_{0}c_{r}^{2}\right)+\frac{1}{k}g^{nn}{\left(\frac{\partial S}{\partial x^{\mu }}\right)}^{2}\\ \; & +\prod_{i=1}^{3}H_{i}^{-\frac{1}{3}}f{\left(\frac{\partial S}{\partial r}\right)}^{2}+\prod_{i=1}^{3}H_{i}^{-\frac{1}{3}}fb+{\tilde{m}}^{2}=0 \end{array}$$

From this equation, we obtain

(19) $$\begin{array}{c} -\frac{1}{f}\left[\left(1+2a_{0}+2c_{0}c_{t}^{2}\right)\right]\prod_{i=1}^{3}H_{i}^{\frac{2}{3}}{\left(\frac{\partial S}{\partial t}-eA_{t}\right)}^{2}+\left(1+2a_{0}+2c_{0}c_{r}^{2}\right)\prod_{i=1}^{3}H_{i}^{-\frac{1}{3}}f{\left(\frac{\partial S}{\partial r}\right)}^{2}+{\tilde{m}}^{2}=\frac{\lambda }{r^{2}} \end{array}$$

(20) $$\begin{array}{c} g^{nn}\frac{\partial S}{\partial x^{n}}+\lambda =0 \end{array}$$

Here, $x^{n}$ represents the three coordinates in five-dimensional spacetime apart from *t* and *r*, specifically $\theta_{1},\theta_{2}$, and $\theta_{3}$. For quantum tunneling radiation, we only need to consider the radial Equation (19). Since this black hole is a ( n+2 )-dimensional static higher-dimensional black hole, it is straightforward to determine its event horizon $r_{H}$ from the zero surface equation given by the following equation:

(21) $$\begin{array}{cc} f\left(r_{H}\right) & =1-\frac{2M}{r_{H}}+\frac{r_{H}^{2}}{l^{2}}\prod_{i=1}^{3}H_{i}=0\\ H_{i} & =1+\frac{q_{i}}{r_{H}^{2}} \end{array}$$

Therefore, at the event horizon of this black hole, the radial Equation (19) simplifies to

(22) $$\begin{array}{c} {\left[f^{2}{\left(\frac{\partial S}{\partial r}\right)}^{2}\right]}_{r\to r_{H}}\prod_{i=1}^{3}H_{i}^{-\frac{1}{3}}\left(1+2a_{0}+2c_{0}c_{r}^{2}\right)-\prod_{i=1}^{3}H_{i}^{\frac{2}{3}}{\left(\frac{\partial S}{\partial t}-eA_{t}\right)}^{2}\left(1+2a_{0}+2c_{0}c_{r}^{2}\right)=0 \end{array}$$

where the action *S* for the spin-$\frac{1}{2}$ fermion in the black hole spacetime can be separated as follows:

(23) $$\begin{array}{c} S=\omega t+R\left(r\right)+Y\left(\theta_{1},\theta_{2},\theta_{3}\right) \end{array}$$

where ω is the energy of the spin-$\frac{1}{2}$ fermion and R(r) is the radial action for the spin-$\frac{1}{2}$ fermion. By substituting Equation (23) into Equation (22), we obtain

(24) $$\begin{array}{c} {\left.\frac{dR}{\;dr}\right|}_{r\to r_{H}}=\pm \frac{1}{{\left.f\right|}_{r\to r_{H}}}{\left(\frac{1+2a_{0}+2c_{0}c_{t}^{2}}{1+2a_{0}+2c_{0}c_{r}^{2}}\right)}^{1/2}\prod_{i=1}^{3}H_{i}^{\frac{1}{2}}\left(\omega -\omega_{0}\right) \end{array}$$

where $\omega_{0}=\frac{e\sqrt{q_{i}\left(q_{i}+2m\right)}}{r_{H}^{2}+q_{i}}$ is the chemical potential and $H_{i}=H_{i}\left(r_{H}\right)$. By applying the residue theorem, we can integrate to obtain

(25) $$\begin{array}{c} R^{\pm }=\pm \frac{i\pi }{f^{\prime }\left(r_{H}\right)}{\left(\frac{1+2a_{0}+2c_{0}c_{t}^{2}}{1+2a_{0}+2c_{0}c_{r}^{2}}\right)}^{1/2}\prod_{i=1}^{3}H_{i}^{\frac{1}{2}}\left(\omega -\omega_{0}\right) \end{array}$$

Let $T_{H}$ represent the Hawking temperature of this black hole. According to WKB theory and quantum tunneling radiation theory, we can obtain the quantum tunneling rate of the spin-$\frac{1}{2}$ fermion at the event horizon of this black hole as

(26) Γ∼exp−2ImR+−ImR−=exp−4πf′rH∏i=13Hi121+2a0+2c0ct21+2a0+2c0cr212ω−ω0=exp−ω−ω0TH

where

(27) $$\begin{array}{c} T_{H}=\frac{f^{\prime }\left(r_{H}\right)}{4\pi }\prod_{i=1}^{3}H_{i}^{-\frac{1}{2}}{\left(\frac{1+2a_{0}+2c_{0}c_{t}^{2}}{1+2a_{0}+2c_{0}c_{r}^{2}}\right)}^{\frac{1}{2}}=T_{h}{\left(\frac{1+2a_{0}+2c_{0}c_{r}^{2}}{1+2a_{0}+2c_{0}c_{t}^{2}}\right)}^{\frac{1}{2}} \end{array}$$

where

(28) $$\begin{array}{c} f^{\prime }\left(r_{H}\right)=2\left[\frac{2M}{r_{H}^{3}}-\frac{1}{l^{2}}\right]\prod_{i=1}^{3}\frac{q_{i}}{r_{H}}+\frac{r_{H}}{l^{2}}\prod_{i=1}^{3}H_{i}\left(r_{H}\right) \end{array}$$

and

(29) $$\begin{array}{c} T_{h}=\frac{f^{\prime }\left(r_{H}\right)}{4\pi }\prod_{i=1}^{3}H_{i}^{-\frac{1}{2}}\left(r_{H}\right) \end{array}$$

where $T_{h}$ is the Hawking temperature without Lorentz breaking corrections. Equation (27) presents the specific expression of the effect of Lorentz breaking on the black hole temperature. For different black holes, the expression of Lorentz breaking’s effect on the black hole temperature varies. The Hawking temperature $T_{H}$ of this black hole is consistent with that presented in reference [46]. Taking into account the Lorentz breaking corrections, the Hawking temperature at the event horizon is modified such that $T_{H}$ is not only related to the coefficient $a_{0}$ of the Lorentz breaking correction term CFJ and the coefficient $c_{0}$ of the Einstein-aether correction term but also depends on the coefficients $c_{t}$ and $c_{r}$ of the components $u^{t}$ and $u^{r}$ of the aether-like field vector. Notably, $T_{H}$ does not depend on the coefficient $b_{0}$ of the chiral correction term, mainly because ${\left(fb\right)|}_{r\to r_{H}}=0$. It should be noted that at the event horizon of this black hole, ${f|}_{r\to r_{H}}=0$, which is why $b_{0}$ does not appear in Equation (27). However, when $r>r_{H}$, the radial action of the spin-1/2 fermion and the particle energy level distributions will be influenced by $b_{0}$. Another significant physical quantity in the context of gauged supergravity theory for a higher-dimensional static multi-charge black hole is the Bekenstein-Hawking entropy. Due to the Lorentz breaking corrections, the entropy associated with the Hawking temperature of this black hole will also experience certain modifications. To fully describe the thermodynamic quantities of this black hole and its corresponding first law of thermodynamics, the thermodynamic quantities of this black hole are given as follows, according to reference [47]:

(30) $$\begin{array}{cc} \; & M=\frac{3\pi }{4}m+\frac{\pi }{4}\sum_{i=1}^{3}q_{i},\;Q_{i}=\frac{\pi }{4}\sqrt{q_{i}\left(q_{i}+2m\right)},\;S=\frac{\pi }{2}\prod_{i=1}^{3}{\left(r_{H}+q_{i}\right)}^{1/2}\\ \; & V=\frac{\pi^{2}r_{h}^{4}}{6}\prod_{i=1}^{3}H_{i}\left(r_{h}\right)\sum_{j=1}^{3}\frac{1}{H_{j}\left(r_{h}\right)},\;\Phi_{i}=\frac{\sqrt{q_{i}\left(q_{i}+2m\right)}}{r_{h}^{2}+q_{i}},\;P=\frac{3}{4\pi l^{2}} \end{array}$$

Another thermodynamic quantity, $T_{h}$, represents the temperature of this black hole as shown in Equation (29). Then one can verify that these seven thermodynamic quantities fully comply with the first law of black hole thermodynamics and the Bekenstein-Smarr mass formula as follows:

(31) $$\begin{array}{c} dM=T_{h}dS_{bh}+\sum_{i=1}^{3}\Phi_{i}dQ_{i}+VdP \end{array}$$

(32) $$\begin{array}{c} 2M=3T_{h}S_{bh}+2\sum_{i=1}^{3}\Phi_{i}dQ_{i}+2VdP \end{array}$$

Here, $T_{h}$, as shown in Equation (29), is the original temperature of this black hole, and the corresponding original Bekenstein-Hawking entropy is denoted as $S_{bh}$. The Lorentz breaking corrections lead to modifications in $S_{bh}$. The modified Bekenstein-Hawking entropy of this black hole is represented as $S_{BH}$. Thus, from Equations (27)–(31), we can obtain

(33) $$\begin{array}{c} S_{BH}=\int \frac{dM-\sum_{i=1}^{3}\Phi_{i}dQ_{i}+VdP}{T_{h}}{\left(\frac{1+2a_{0}+2c_{0}c_{t}^{2}}{1+2a_{0}+2c_{0}c_{r}^{2}}\right)}^{\frac{1}{2}}=S_{bh}{\left(\frac{1+2a_{0}+2c_{0}c_{t}^{2}}{1+2a_{0}+2c_{0}c_{r}^{2}}\right)}^{\frac{1}{2}} \end{array}$$

Here, $S_{bh}=\frac{\pi^{2}}{2}\prod_{i=1}^{3}{\left(r_{h}^{2}+q_{i}\right)}^{1/2}$ is the original Bekenstein-Hawking entropy of this black hole. From Equation (33), it can be seen that the coefficients of the Lorentz breaking correction term CFJ and the Einstein-aether term have a significant impact on the entropy of this black hole. The entropy $S_{BH}$, modified by the Lorentz breaking corrections, represents a meaningful new expression. $S_{BH}$ is derived within the framework of semiclassical theory. In fact, from Equations (7) and (12), it is clear that terms containing *ℏ* have been neglected; therefore, both of these equations and the conclusions drawn from them are results relevant to semiclassical theory. To investigate the corrections from quantum perturbation theory to the above semiclassical results, we need to let $\tilde{\omega_{0}}=\omega -\omega_{0}$ and express the particle energy and action in terms of *ℏ* perturbations as

(34) $$\begin{array}{cc} \tilde{\omega } & =\tilde{\omega_{0}}+\sum_{k=1}\hbar^{k}\tilde{\omega^{k}} \end{array}$$

(35) $$\begin{array}{cc} {\tilde{R}}^{\pm } & =R_{0}^{\pm }+\sum_{k=1}\hbar^{k}\tilde{R_{k}} \end{array}$$

$R_{0}$ is the radial action in the semiclassical theory of particles. The equations satisfied by $R_{0}^{\pm }$ and $\tilde{\omega_{0}}$ can be expressed based on Equation (24) as follows:

(36) $$\begin{array}{c} {\left.{\left(\frac{dR_{0}}{\;dr}\right)}^{2}\right|}_{r\to r_{H}}=\prod_{i=1}^{3}H_{i}\left(\frac{1+2a_{0}+2c_{0}c_{t}^{2}}{1+2a_{0}+2c_{0}c_{r}^{2}}\right)\frac{{\tilde{\omega_{0}}}^{2}}{{\left.f^{2}\right|}_{r\to r_{H}}} \end{array}$$

When we take into account the *ℏ* perturbation, based on Equations (22) and (36), we can obtain the following equation:

(37) $$\begin{array}{cc} \; & {\left.{\left(\frac{d\tilde{R_{1}}}{\;dr}\right)}^{2}\right|}_{r\to r_{H}}=\prod_{i=1}^{3}H_{i}\left(\frac{1+2a_{0}+2c_{0}c_{t}^{2}}{1+2a_{0}+2c_{0}c_{r}^{2}}\right)\frac{{\tilde{\omega_{1}}}^{2}}{{\left.f^{2}\right|}_{r\to r_{H}}} \end{array}$$

(38) $$\begin{array}{cc} \; & {\left.{\left(\frac{d\tilde{R_{2}}}{\;dr}\right)}^{2}\right|}_{r\to r_{H}}=\prod_{i=1}^{3}H_{i}\left(\frac{1+2a_{0}+2c_{0}c_{t}^{2}}{1+2a_{0}+2c_{0}c_{r}^{2}}\right)\frac{{\tilde{\omega_{2}}}^{2}}{{\left.f^{2}\right|}_{r\to r_{H}}}\\ \; & \vdots \end{array}$$

Therefore, from ${\tilde{R}}^{\pm }=R_{0}^{\pm }+\hbar R_{1},R_{0}^{\pm }+\hbar R_{1}+\hbar^{2}R_{2},\ldots$, it can be seen that there is an inherent relationship between $R_{k+1}$ and $R_{k}$. By letting $\frac{{\tilde{R}}_{k}^{\pm }}{{\tilde{R}}_{k+1}^{\pm }}=\frac{a_{k}}{S_{BH}}$, we can express the radial component of the particle action after the *ℏ* perturbation correction ${\tilde{R}}^{\pm }$ specifically, as shown in Equation (35):

(39) $$\begin{array}{c} {\tilde{R}}^{\pm }=\pm \frac{i\pi }{f^{\prime }\left(r_{H}\right)}{\left(\frac{1+2a_{0}+2c_{0}c_{t}^{2}}{1+2a_{0}+2c_{0}c_{r}^{2}}\right)}^{\frac{1}{2}}\prod_{i=1}^{3}H_{i}^{\frac{1}{2}}\left(1+\sum_{k=1}\frac{\hbar^{k}a_{k}}{S_{BH}}\right)\left(\omega -\omega_{0}\right) \end{array}$$

The quantum tunneling rate of this black hole has been further corrected to

(40) Γ∼exp−2ImR+˜−ImR−˜=exp−ω−ω0TH˜

where

(41) $$\begin{array}{c} \tilde{T_{H}}=T_{H}{\left(1+\sum_{k=1}\frac{h^{k}a_{k}}{S_{BH}}\right)}^{-1} \end{array}$$

$\tilde{T_{H}}$ is the black hole temperature that incorporates both Lorentz breaking corrections and quantum corrections. The first law of black hole thermodynamics can be restated as follows:

(42) $$\begin{array}{c} dM=\tilde{T_{H}}dS_{bh}+\sum_{i=1}^{3}\Phi_{i}dQ_{i}+VP \end{array}$$

From this, we can obtain the Bekenstein-Hawking entropy of this black hole after Lorentz breaking corrections and quantum corrections, denoted as $\tilde{S_{BH}}$, as follows:

(43) $$\begin{array}{cc} \tilde{S_{BH}} & =\int \frac{dM-\sum_{i=1}^{3}\Phi_{i}dQ_{i}-VP}{\tilde{T_{H}}}=\int \frac{dM-\sum_{i=1}^{3}\Phi_{i}dQ_{i}-VP}{T_{H}}\left(1+\sum_{k=1}\frac{\hbar^{k}a_{k}}{S_{BH}}\right)\\ \; & =S_{BH}+\hbar a_{1}\ln S_{BH}+\cdots \end{array}$$

Here, we obtain a new expression for the Bekenstein-Hawking entropy of static multi-charge AdS black holes in the context of gauged supergravity theories. The expression $\tilde{S_{BH}}$ includes the results of Lorentz breaking and quantum perturbation corrections. From the above $S_{bh},S_{BH}$, and $\tilde{S_{BH}}$, it is clear that black hole thermodynamics is a topic worth in-depth study. As the quantum tunneling radiation of the black hole progresses, the physical quantities such as the temperature and mass of the black hole will change, and the entropy of the black hole will also change. As the black hole evolves, we can denote the change in the Bekenstein-Hawking entropy of this black hole as $\Delta \tilde{S_{BH}}$. The quantum tunneling rate, as shown in Equation (40), can be further expressed as $\Gamma =\exp\left(\Delta \tilde{S_{BH}}\right)$. This represents the corrected result for the entropy of five-dimensional static multi-charge AdS black holes in gauged supergravity theories.

The spin-1/2 fermion, also known as the Dirac particle, has a wave function $\psi_{1/2}$ that satisfies the Dirac equation $\left(\gamma^{\mu }D_{\mu }+\frac{m}{\hbar }\right)\psi_{1/2}=0$ in curved spacetime. When considering Lorentz invariance violation, appropriate modifications need to be made to the Dirac equation. Specifically, a correction term containing a coupling correction coefficient σ(σ<<1) must be added to the original Dirac equation, resulting in the term $\sigma \hbar \gamma^{t}\gamma^{\phi }D_{t}D_{\phi }\psi_{1/2}$. Similarly, by choosing the Hermitian matrices $\gamma^{\mu }$, the modified Dirac equation accounting for Lorentz invariance violation can be used to study the corrected results for black hole entropy.

## 4. Discussion

We first modified the action of spin-1/2 fermion in higher-dimensional curved spacetime. Based on the characteristics of higher-dimensional curved spacetime, we studied the gamma matrices in the matrix equation describing the spin-1/2 fermion wave function. Starting from the dynamics of the Lorentz breaking modified spin-1/2 fermion, we examined the quantum tunneling rate, Hawking temperature at the event horizon, Bekenstein-Hawking entropy, and their evolutionary characteristics of a black hole in the context of a five-dimensional static multi-charge AdS black hole within a gauge supergravity theory. By correctly selecting the corresponding gamma matrices $\gamma^{\mu }$ and Einstein-aether vector $u^{\mu }$ for this (n+2)-dimensional spacetime (n>2), we obtained the quantum tunneling rate and Hawking temperature at the event horizon for this specific type of higher-dimensional static multi-charge black hole in supergravity theory. On this basis, according to the first law of black hole thermodynamics and the complete thermodynamic quantities of the black hole, we also derived a new expression for the Bekenstein-Hawking entropy of this black hole. Building upon the results obtained from these semiclassical theories, we further investigated a new expression for the corrected entropy of this black hole based on quantum perturbation theory. In this series of results, all are related to $q_{i}$, where i=1,2,3. Therefore, all three expressions for the black hole entropy have connections to $q_{i}$. This is a fundamental characteristic of the five-dimensional three-charge AdS black hole. Different conclusions can be drawn based on the different cases of $q_{i}$, such as $q_{1}\neq 0,q_{2}=q_{3}=0$, which corresponds to the K-K gauged supergravity case; $q_{1}=q_{2}\neq 0,q_{3}=0$, which refers to the EMDA gauged supergravity case; $q_{1}\neq q_{2}\neq 0,q_{3}=0$, representing the case of the five-dimensional AdS Horowitz-Sen solution; $q_{1}=q_{2}=q_{3}\neq 0$, which corresponds to the RN-${\mathrm{AdS}}_{5}$ case; and $q_{1}\neq q_{2}\neq q_{3}\neq 0$, representing the STU gauged supergravity case. For the five-dimensional static multi-charged AdS black hole, the physical significance of the series of results obtained through various calculations is rich. It is important to note that for higher-dimensional static multi-charged black holes, the research methods used and the results obtained have certain reference significance. The above methods cannot be simply applied to dynamic curved spacetimes or higher-dimensional static curved spacetimes; instead, the gamma matrices $\gamma^{\mu }$ and $\gamma^{5}$ must be constructed correctly based on the specific characteristics of the curved spacetime. For spin-3/2, spin-5/2,…, additional conditions must be imposed to conduct research. The modification of dynamical characteristics of scalar particles in higher-dimensional curved spacetimes cannot be studied using the above methods. In summary, this paper provides a method for studying the corrected black hole entropy in higher-dimensional static curved spacetimes and its related results. By combining the topological classes of the thermodynamics of static multi-charge AdS black holes in gauged supergravity [48], we can deepen our understanding of related topics in higher-dimensional spacetimes.

## Data Availability

Data is contained within the article.
